# Supplementary material for: Inter-Rater Agreement in Assessing Risk of Bias in Melanoma Prediction Studies Using the Prediction Model Risk of Bias Assessment Tool (PROBAST): Results from a Controlled Experiment on the Effect of Specific Rater Training
Source: J Clin Med. 2023 Mar 2;12(5):1976. doi: 10.3390/jcm12051976 (PMC10003882; doi:10.3390/jcm12051976)
Supplement: Supplementary file 1 [file jcm-12-01976-s001.zip › jcm-2203054-supplementary.pdf]

Supplement

Table S1: Number of low, high, and unclear ROB ratings, as well as consensus decisions per PROBAST domain and overall for all included studies. Studies are divided into "studies rated before training" and "studies rated after training", and sorted by the last name of the first author. (N=42)

	Domain "participants"				Domain "predictors"				Domain "outcome"				Domain "analysis"				Overall ROB			
	+	-	?	Consensus	+	-	?	Consensus	+	-	?	Consensus	+	-	?	Consensus	+	-	?	Consensus
**Studies rated before training**																				
Augustsson et al. [31]	5	0	1	+	6	0	0	+	5	0	1	+	0	4	2	-	1	4	1	-
Barbini et al. [34]	2	2	2	-	5	0	1	+	3	0	3	+	0	0	6	?	2	3	1	-
Cho et al. [36]	2	1	3	+	4	1	1	+	2	0	4	-	4	0	2	+	0	2	4	-
Cust et al. [38]	5	0	1	+	3	0	3	?	5	0	1	+	3	0	3	+	3	0	3	?
Davies et al. [39]	1	2	3	-	2	1	3	+	1	0	5	+	4	1	1	+	2	0	4	-
Dwyer et al. [40]	6	0	0	+	4	0	2	+	6	0	0	+	1	3	2	-	2	3	1	-
Fang et al. [42]	2	1	3	-	3	0	2	?	2	0	4	+	4	1	1	+	2	0	4	-
Fears et al. [44]	4	1	1	-	4	0	2	?	5	0	1	+	1	1	4	-	2	2	2	-
Fortes et al. [46]	4	2	0	-	3	0	3	?	6	0	0	+	1	2	3	+	2	3	1	-
Gu et al. [50]	3	2	1	-	3	1	2	-	3	0	3	+	3	0	3	?	2	2	2	-
Guther et al. [51]	2	3	1	-	5	0	1	+	3	0	3	-	2	2	2	?	1	3	2	-
Landi et al. [55]	5	1	0	-	4	0	2	?	6	0	0	+	1	3	2	-	2	3	1	-
Marett et al. [58]	6	0	0	+	4	0	2	?	5	0	1	+	1	3	2	-	2	3	1	-
Nielsen et al. [59]	5	1	0	+	4	0	2	+	6	0	0	+	2	3	1	-	2	4	0	-
Nikolic et al. [60]	2	2	2	-	3	1	2	?	3	0	3	+	2	2	2	?	1	3	2	-
Olsen et al. [61]	6	0	0	+	5	0	1	+	6	0	0	+	5	0	1	+	6	0	0	+
Stefanaki et al. [67]	5	1	0	-	5	0	1	?	5	0	1	+	1	3	2	-	3	3	0	-
Tagliabue et al. [68]	0	3	3	-	1	3	2	-	2	1	3	+	1	3	2	-	0	5	1	-
Vuong et al. [70]	5	1	0	+	3	1	2	?	4	0	2	+	5	0	1	+	3	2	1	?
Williams et al. [72]	6	0	0	+	4	1	1	?	4	0	2	+	3	0	3	?	2	3	1	?
**Studies rated after training**																				
Bakos et al. [32]	2	3	1	-	1	2	3	?	6	0	0	+	1	4	1	-	1	5	0	-
Bakshi et al. [33]	6	0	0	+	5	0	1	+	3	2	1	+	2	2	2	-	2	2	2	-
Cho et al. [35]	3	1	2	-	1	4	1	-	6	0	0	+	4	2	0	+	1	4	1	-
Cust et al. [37]	5	0	1	+	3	0	3	?	6	0	0	+	6	0	0	+	3	0	3	?
English and Armstrong [41]	6	0	0	+	2	0	4	?	5	0	1	+	2	1	3	?	0	1	5	?

Fargnoli et al. [43]	1	5	0	-	2	0	4	?	6	0	0	+	0	5	1	-	0	6	0	-
Fontanillas et al. [45]	3	2	1	?	3	0	3	?	1	3	2	-	5	0	1	+	2	3	1	-
Garbe et al. [48]	1	5	0	-	1	1	5	?	5	0	1	+	0	5	1	-	0	6	0	-
Garbe et al. [47]	3	3	0	-	3	0	3	?	6	0	0	+	0	5	1	-	0	6	0	-
Goldberg et al. [49]	5	1	0	-	5	0	1	+	0	4	2	-	1	5	0	-	0	6	0	-
Harbauer et al. [52]	1	5	0	-	4	0	2	?	6	0	0	+	0	5	1	-	0	5	1	-
Hübner et al. [53]	4	1	1	-	5	0	1	+	6	0	0	+	1	3	2	-	1	3	2	-
Kypreou et al. [54]	1	5	0	-	6	0	0	+	6	0	0	+	5	0	1	+	1	5	0	-
MacKie et al. [56]	1	5	0	-	0	1	5	?	6	0	0	+	0	4	2	-	0	6	0	-
Mar et al. [57]	1	4	1	-	0	2	4	?	4	0	2	+	0	5	1	-	0	6	0	-
Penn et al. [62]	5	0	1	+	2	0	4	?	6	0	0	+	4	0	2	?	2	1	3	?
Quéreux et al. [63]	1	5	0	-	3	0	3	?	6	0	0	+	2	3	1	+	0	6	0	-
Richter and Koshgoftaar [64]	2	4	0	-	2	0	4	?	6	0	0	+	1	2	3	?	0	5	1	-
Smith et al. [65]	1	0	5	?	2	1	3	?	4	0	2	?	0	1	5	-	0	3	3	-
Sneyd et al. [66]	6	0	0	+	4	0	2	?	6	0	0	+	3	0	3	+	2	3	1	-
Vuong et al. [69]	5	0	1	+	3	0	3	?	6	0	0	+	5	0	1	+	2	3	1	?
Whiteman and Green [71]	0	0	6	?	1	1	4	?	4	0	2	+	0	2	4	-	0	3	3	-

Abbreviations: PROBAST = Prediction model Risk Of Bias ASsessment Tool; ROB = risk of bias; + indicates low ROB; - indicates high ROB; ? indicates unclear ROB

	Conger's Kappa before training	Conger's Kappa after training	Difference (95% CI)
Participants	0.165	0.391	0.226 (0.032, 0.407)
Predictors	0.0262	0.154	0.128 (−0.010, 0.256)
Outcome	0.181	0.295	0.114 (−0.166, 0.367)
Analysis	0.131	0.277	0.146 (−0.020, 0.295)
Overall	0.099	0.257	0.157 (−0.016, 0.323)

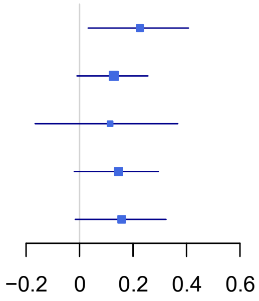

Figure S1: Multi-rater agreement in terms of Conger's κ before and after training for the domain-specific and overall ROB rating, as well as difference in agreement with bootstrapped 95%-CI.

Table S2: Pairwise inter-rater agreements in terms of AC_1 estimates for the ROB rating in the domain "participants".

		Rater 1	Rater 2	Rater 3	Rater 4	Rater 5	Rater 6
**Rater 1**	Before<br>After	-	-	-	-	-	-
**Rater 2**	Before<br>After	0.453<br>0.669	-	-	-	-	-
**Rater 3**	Before<br>After	0.462<br>0.673	0.530<br>0.543	-	-	-	-
**Rater 4**	Before<br>After	0.337<br>0.670	0.620<br>0.539	0.349<br>0.935	-	-	-
**Rater 5**	Before<br>After	0.481<br>0.212	0.559<br>0.212	0.424<br>0.423	0.634<br>0.353	-	-
**Rater 6**	Before<br>After	0.357<br>0.538	0.040<br>0.471	-0.011<br>0.406	-0.017<br>0.403	0.141<br>-0.086	-

Table S3: Pairwise inter-rater agreements in terms of AC_1 estimates for the ROB rating in the domain "predictors".

		Rater 1	Rater 2	Rater 3	Rater 4	Rater 5	Rater 6
**Rater 1**	Before<br>After	-	-	-	-	-	-
**Rater 2**	Before<br>After	0.275<br>0.808	-	-	-	-	-
**Rater 3**	Before<br>After	0.164<br>0.368	-0.028<br>0.163	-	-	-	-
**Rater 4**	Before<br>After	0.232<br>0.301	0.625<br>0.096	0.058<br>0.940	-	-	-
**Rater 5**	Before<br>After	0.416<br>0.289	0.718<br>0.088	-0.024<br>0.178	0.633<br>0.235	-	-
**Rater 6**	Before<br>After	0.278<br>0.399	0.119<br>0.523	0.315<br>-0.069	0.208<br>-0.002	0.264<br>0.135	-

Table S4: Pairwise inter-rater agreements in terms of AC_1 estimates for the ROB rating in the domain "outcome".

		Rater 1	Rater 2	Rater 3	Rater 4	Rater 5	Rater 6
**Rater 1**	Before<br>After	-	-	-	-	-	-
**Rater 2**	Before<br>After	0.620<br>0.623	-	-	-	-	-
**Rater 3**	Before<br>After	0.393<br>0.903	0.534<br>0.671	-	-	-	-
**Rater 4**	Before<br>After	0.353<br>0.903	0.620<br>0.671	0.461<br>1.000	-	-	-
**Rater 5**	Before<br>After	0.754<br>0.848	0.643<br>0.660	0.346<br>0.742	0.508<br>0.742	-	-
**Rater 6**	Before<br>After	0.747<br>0.797	0.631<br>0.772	0.400<br>0.845	0.241<br>0.845	0.762<br>0.623	-

Table S5: Pairwise inter-rater agreements in terms of AC_1 estimates for the ROB rating in the domain "analysis".

		Rater 1	Rater 2	Rater 3	Rater 4	Rater 5	Rater 6
**Rater 1**	Before<br>After	-	-	-	-	-	-
**Rater 2**	Before<br>After	0.502<br>0.735	-	-	-	-	-
**Rater 3**	Before<br>After	-0.146<br>0.339	-0.159<br>0.407	-	-	-	-
**Rater 4**	Before<br>After	-0.049<br>0.335	0.120<br>0.340	0.283<br>0.738	-	-	-
**Rater 5**	Before<br>After	0.062<br>-0.090	0.249<br>-0.090	0.056<br>-0.015	0.184<br>-0.021	-	-
**Rater 6**	Before<br>After	0.568<br>0.531	0.335<br>0.667	0.112<br>0.273	0.105<br>0.334	-0.100<br>-0.019	-

Table S6: Pairwise inter-rater agreement in terms of AC_1 estimates for all pairs of raters for the overall ROB rating.

		Rater 1	Rater 2	Rater 3	Rater 4	Rater 5	Rater 6
**Rater 1**	Before<br>After	-	-	-	-	-	-
**Rater 2**	Before<br>After	0.596<br>0.754	-	-	-	-	-
**Rater 3**	Before<br>After	0.057<br>0.569	0.114<br>0.447	-	-	-	-
**Rater 4**	Before<br>After	-0.102<br>0.568	-0.167<br>0.384	-0.075<br>0.815	-	-	-
**Rater 5**	Before<br>After	-0.265<br>0.166	-0.260<br>0.167	-0.083<br>0.230	0.486<br>0.422	-	-
**Rater 6**	Before<br>After	0.873<br>0.654	0.603<br>0.655	0.140<br>0.423	-0.250<br>0.537	-0.190<br>0.331	-
